# Quo vadis, Kras?

**DOI:** 10.18632/oncotarget.1322

**Published:** 2013-08-22

**Authors:** Günter Schneider, Dieter Saur

**Affiliations:** Technische Universität München, Klinikum rechts der Isar, II. Medizinische Klinik, Ismaninger Str. 22, 81675 Munich, Germany; Technische Universität München, Klinikum rechts der Isar, II. Medizinische Klinik, Ismaninger Str. 22, 81675 Munich, Germany

Somatic mutations in the *Ras* oncogene family (*KRas*, *HRas*, and *NRas*) occur in up to 30% of human cancers. Due to this high mutation frequency and the recent observation that survival of Kras^G12D^-driven cancer cells depends on the continuous expression of the oncogene *in vivo* [1, 2], mutant Kras is an excellent therapeutic target. However, efforts to develop drugs, which directly target mutant Kras failed in the past and remain an ambitious challenge for future research. Therefore, inhibition of essential downstream effectors of oncogenic Kras offer an alternative road to fight against Kras addicted cancers. Prerequisite for such an approach is the definition of crucial Kras engaged driver and effector pathways in preclinical cancer models. Signaling induced by oncogenic Kras flux through three main branches: the canonical Raf-Mek-Erk, the Phosphatidylinositol 3-kinase (PI3K) and the Ral guanine nucleotide exchange factor pathway. Pancreatic ductal adenocarcinoma (PDAC) and non-small cell lung cancer (NSCLC) harbor Kras mutations with high frequency. Recently, the role of Kras effectors for cancer initiation and development has been clarified using genetic gain and loss of function models *in vivo*. Whereas Craf is essentially involved in the development of Kras-driven lung cancer [3], it is dispensable for Kras-dependent pancreatic carcinogenesis [4]. Although this does not exclude the contribution of canonical Ras signaling for pancreatic carcinogenesis, indeed oncogenic Braf^V600E^ is even more aggressive than oncogenic Kras to induce preneoplastic lesions and cancer in the pancreas (unpublished results and [5]), it impressively demonstrates that signaling components needed for Kras-driven cancer development are highly tissue and context specific. Consistent with this concept, signaling modules of the PI3K pathway influence Kras-driven carcinogenesis in the pancreas and the lung differentially. Phosphatidylinositol 3-kinases, like the class IA catalytical subunit p110α, phosphorylate the 3' hydroxyl group of phosphatidylinositols to generate the second messenger phophatidylinositol (3,4,5) trisphosphate (PIP3). PIP3 recruits the AGC kinase family member's 3-phosphoinositide-dependent protein kinase 1 (PDK1) and AKT to the plasma membrane, where PDK1 activates AKT by Threonin 308 phosphorylation. Expression of an oncogenic p110α mutant, p110α^H1047R^, in the pancreatic epithelium phenocopies Kras^G12D^-driven carcinogenesis with striking similarity [4]. Accordingly, conditional deletion of the PI3K effector *PDK1* in the pancreas completely blocks Kras^G12D^-driven preneoplastic lesion and cancers, demonstrating an essential contribution of PI3K signaling in Kras-dependent pancreatic carcinogenesis [4]. In contrast to the pancreas, PDK1 is dispensable for Kras^G12D^-induced lung cancer development [4], corroborating the notice of context and tissue specific effector components of oncogenic Kras. Although it will be a great goal in future research to find the molecular hubs controlling these tissue and context specific requirements during carcinogenesis, it will also be important to prove whether the concept of tissue and context specific effectors of oncogenic Kras is valid for tumor maintenance. Recent *in vitro* work with a large cell line platform representing common human tumor entities revealed that Kras mutations correlate with increased sensitivity towards MEK inhibitors and a decreased sensitivity towards inhibitors of the PI3K pathway [6]. This argues that common molecular programs are directed by oncogenic Kras to secure tumor maintenance. Consistent with this *in vitro* observation, Kras^G12D^-driven murine lung cancers are relatively refractory towards PI3K inhibitor treatment *in vivo* [7]. In contrast, murine PDAC cells are equally sensitive towards PI3K inhibitors, irrespectively whether initiated and driven by Kras^G12D^ or p110α^H1047R^ [8]. Furthermore, PI3K inhibitors prevent tumor progression in Kras^G12D^-dependent mouse models and humanized primary patient derived orthotopic xenotransplantation models of PDAC [4]. This clearly demonstrates the importance of the PI3K pathway for tumor maintenance in the pancreas. Especially the *in vivo* data support the idea of tissue- and context-specific signaling components, which are engaged by oncogenic Kras to maintain tumors. However, to prove such a concept, we need *in vivo* evidence at the level of genetics. Therefore, there is an urgent demand to create novel *in vivo* cancer models, allowing tissue- and time-specific activation or inactivation of genes and signaling pathways in established tumors. Such models rely for example on the use of two or more recombination systems to achieve the goal of time- and tissue-specificity. They are currently under development in several laboratories worldwide and will certainly help to define important tumor entity specific therapeutic targets. In addition, global deletion of Kras effectors in the whole animal will allow prediction of the in vivo toxicity of potential inhibitors, an important aspect for translational research. The proof of tumor- and tissues-specific Kras effector's in the tumor maintenance program would have immediate impact on clinical practice, since it would demonstrate that there is no magic bullet for all tumor entities initiated, driven and maintained by oncogenic Kras. Instead, research would have to determine tumor entity specific Kras-activated signaling components, which are essential and non-redundant.

Also 30 years after the description of the human Ras oncogenes, cure for most Ras-driven cancers is not available. However, new sophisticated tumor models allow definition of essential Ras effectors in a tumor entity specific context. Thus, novel and important target's with the potential to offer cure emerge for Kras-driven tumor entities. Therefore, we are confident that science will improve the outcome of Ras-mutated tumors in the next decade.
